# Neuroprotective effect of Src kinase in hypoxia-ischemia: A systematic review

**DOI:** 10.3389/fnins.2022.1049655

**Published:** 2022-11-24

**Authors:** Panagiotis Christidis, Abhya Vij, Stamatios Petousis, Javid Ghaemmaghami, Bhairav V. Shah, Ioannis Koutroulis, Panagiotis Kratimenos

**Affiliations:** ^1^Laboratory of Physiology, Faculty of Health Sciences, School of Medicine, Aristotle University of Thessaloniki, Thessaloniki, Greece; ^2^Department of Pediatrics, Boston Children's Hospital and Harvard Medical School, Boston, MA, United States; ^3^2nd Department of Obstetrics and Gynecology, “Hippokrateion” General Hospital of Thessaloniki, Aristotle University of Thessaloniki, Thessaloniki, Greece; ^4^Center for Neuroscience Research, Children's National Research Institute, Washington, DC, United States; ^5^Division of Pediatric Surgery, Department of Pediatrics, School of Medicine, Prisma Health Children's Hospital-Midlands, University of South Carolina, Columbia, SC, United States; ^6^Department of Pediatrics, Division of Emergency Medicine, Children's National Hospital, George Washington University School of Medicine and Health Sciences, Washington, DC, United States; ^7^Division of Neonatology, Department of Pediatrics, Children's National Hospital, George Washington University School of Medicine and Health Sciences, Washington, DC, United States

**Keywords:** Src, hypoxic-ischemic encephalopathy (HIE), hypoxia, neonatal brain, neuroprotection

## Abstract

**Background:**

Hypoxic-ischemic encephalopathy (HIE) is a major cause of neonatal morbidity and mortality worldwide. While the application of therapeutic hypothermia has improved neurodevelopmental outcomes for some survivors of HIE, this lone treatment option is only available to a subset of affected neonates. Src kinase, an enzyme central to the apoptotic cascade, is a potential pharmacologic target to preserve typical brain development after HIE. Here, we present evidence of the neuroprotective effects of targeting Src kinase in preclinical models of HIE.

**Methods:**

We performed a comprehensive literature search using the National Library of Medicine's MEDLINE database to compile studies examining the impact of Src kinase regulation on neurodevelopment in animal models. Each eligible study was assessed for bias.

**Results:**

Twenty studies met the inclusion criteria, and most studies had an intermediate risk for bias. Together, these studies showed that targeting Src kinase resulted in a neuroprotective effect as assessed by neuropathology, enzymatic activity, and neurobehavioral outcomes.

**Conclusion:**

Src kinase is an effective neuroprotective target in the setting of acute hypoxic injury. Src kinase inhibition triggers multiple signaling pathways of the sub-membranous focal adhesions and the nucleus, resulting in modulation of calcium signaling and prevention of cell death. Despite the significant heterogeneity of the research studies that we examined, the available evidence can serve as proof-of-concept for further studies on this promising therapeutic strategy.

## Introduction

One in four perinatal deaths is attributed to hypoxic-ischemic (HI) brain injury, a term that describes acute interruptions in oxygenated blood flow to the brain (Lawn et al., 2005; Pauliah et al., 2013). Following the perinatal asphyxiation insult, HI involves a cascade of biochemical events that cause cerebral edema, inflammation, neuronal cell injury, and ultimately, neuronal cell death over a period of hours to days (Gunn and Thoresen, 2006; Cilio and Ferriero, 2010; Juul and Ferriero, 2014; Hagberg et al., 2015; Van Bel and Groenendaal, 2016; Delivoria-Papadopoulos et al., 2018). While there have been improvements in survival rates after neonatal HI, many of these patients suffer ongoing neurological impairments that both lessen quality of life and incur burdensome healthcare costs (Blencowe et al., 2013; Eunson, 2015).

The only evidence-based treatment currently available for neonatal HI is therapeutic hypothermia (TH). This approach is targeted at modulating the deleterious cytotoxic and inflammatory processes that occur during HI by tightly regulating temperature (Wyatt et al., 2007), blood pressure, ventilation, and glucose metabolism; for some patients, application of TH has resulted in improved neurological outcomes (Filan et al., 2006; Tam et al., 2012; Wong et al., 2013).

While conceptually promising, the application of TH has several limitations. First, it is mainly available in high-resource countries. Moreover, the protocol requires that total body cooling be initiated within 6 h of birth, leaving clinicians with a narrow window to establish the diagnosis, assess the severity of HI and implement treatment with TH. This time is further compressed for centers that do not have the necessary advanced equipment, staffing, monitoring and experience to provide neonatal TH, as attempts are made to transfer the patient to a suitable facility (Olsen et al., 2013). Finally, despite the use of TH, both the overall mortality rates and the disability rates following application of TH after neonatal HI in published trials remain high (Shankaran et al., 2012).

Most strikingly, even when TH is timely applied in an advanced neonatal intensive care unit, only a subset of neonates with HI have been shown to benefit (Gunn and Thoresen, 2006). Thus, there is a critical and urgent need to develop additional therapeutic strategies that address both morbidity and mortality following neonatal HI. To this end, multiple pre-clinical *in vivo* studies have focused primarily on (a) elucidating the molecular biology underlying HI, (b) identifying potential molecular targets in pathways integral to cerebral injury, (c) optimizing cooling strategies, and (d) recognizing adjuvant therapies that could augment the neuroprotective effects of TH (Jacobs et al., 2013). These studies implicate potential targets of the apoptotic cascade that may, when modulated by pharmacological intervention, offer additional or alternative therapies for HI, critical in cases where TH is not available, particularly in low-resource countries (Robertson et al., 2008; Pauliah et al., 2013; Montaldo et al., 2015), and in treatment of patients who do not respond or respond insufficiently to TH.

In particular, these studies have shown that Src kinase is involved in numerous activated intracellular pathways during HI (Paul et al., 2001; Mishra et al., 2009; Haass and Mandelkow, 2010; Ittner et al., 2010; Delivoria-Papadopoulos et al., 2011; Liu and Sharp, 2011; Hossain et al., 2012; Angelis and Delivoria-Papadopoulos, 2017a,b; Kratimenos et al., 2017). However, there is conflicting evidence regarding its regulatory role, which may differ depending upon brain maturation. Hossain et al. demonstrated that Src kinase activation improves neuronal survival in primary cortical cell cultures (Paul et al., 2001; Haass and Mandelkow, 2010; Ittner et al., 2010; Liu and Sharp, 2011; Hossain et al., 2012), whereas several other studies have shown that Src kinase phosphorylation causes neuronal damage in ischemic stroke, intracerebral hemorrhage, and Alzheimer's disease (Haass and Mandelkow, 2010; Ittner et al., 2010; Liu and Sharp, 2011; Hossain et al., 2012). Porcine experimental models have been used to examine the deleterious effects of Src kinase in neonatal HI and have shown that it can induce the production of free radicals, causing secondary inflammation and excitotoxicity (Kratimenos et al., 2017, 2018, 2022).

Selective Src inhibitors (Src-i) exhibit effectiveness against neuronal cell injury in neonatal and developing animal models and offered neuroprotection as demonstrated by histologic, biochemical, and neurobehavioral assessments (Mishra et al., 2009; Delivoria-Papadopoulos et al., 2011; Angelis and Delivoria-Papadopoulos, 2017a,b; Kratimenos et al., 2017). Contrary to those results, experiments in adult mice showed that Src kinase inhibition worsens cerebral injury (Wang et al., 2004; Guo et al., 2006; Wu et al., 2008; Hu et al., 2009; Tian et al., 2009). In addition, several studies have highlighted the role of Src kinase in neuronal survival after ischemia/reperfusion (I/R) through interactions with the extracellular signal-regulated kinase (ERK) (Wang et al., 2004; Guo et al., 2006; Wu et al., 2008; Hu et al., 2009; Tian et al., 2009).

This systematic review aims to examine the current knowledge regarding the role of Src kinase in neonatal HI and the potential neuroprotective effects of selective Src manipulation.

## Materials and methods

### Protocol

A review of relevant preclinical studies was performed to summarize the current knowledge regarding the role of Src kinase inhibition and its potential benefits on the neonatal hypoxic-ischemic brain. We utilized the CAMARADES (Collaborative Approach to Meta-Analysis and Review of Animal Data from Experimental Studies) guidelines in the methodology (De Vries et al., 2015).

### Literature search

A literature search for the Medline electronic database for all studies up to July 01, 2022. Below, the search strategy for the database is presented: *(((“hypoxia”[MeSH Terms] OR “hypoxia”[All Fields]) OR (“ischaemia”[All Fields] OR “ischemia”[MeSH Terms] OR “ischemia”[All Fields])) OR ((“cerebrum”[MeSH Terms] OR “cerebrum”[All Fields] OR “cerebral”[All Fields] OR “brain”[MeSH Terms] OR “brain”[All Fields]) OR (“brain”[MeSH Terms] OR “brain”[All Fields]))) AND Src kinase[tiab]*. The reference lists of the retrieved articles were subsequently manually reviewed to identify any additional studies that would be considered for inclusion.

### Inclusion criteria

Preclinical studies were included. For inclusion, experimental study protocols were required to involve animals treated with any kind of Src kinase inhibitor before or after the induction of HI. The outcome measures of the studies analyzed were required to include “neuroprotection” defined by histologic, biochemical, and/or neurobehavioral findings.

The current review defines HI as an acute interruption of blood flow and oxygen to the brain. In preclinical animal models, HI is typically induced *via* ligation or occlusion of the common carotid artery or by decreasing oxygen concentration in mechanically ventilated animals. Given the bilaterality of neonatal HI, we focused on experimental protocols that induced global brain hypoxia rather than unilateral hypoxia. Global transient hypoxia could be induced by four-vessel occlusion (4VO) of both vertebral arteries and common carotid arteries, by bilateral occlusion of common carotid arteries (2VO), or by titration of the FiO_2_ below 0.21 (drop of FiO2 to 0.05–0.006 within 5 min, maintained for the 60 min period and titrated to achieve a 40 % reduction in systolic BP from baseline) in a controlled environment for a period of time (Traystman, 2003).

### Exclusion criteria

We excluded all studies that were based on cell lines and *in vitro* experiments. Articles written in languages other than English were also excluded. We also excluded studies that treated animals with unilateral ligation of one of the carotid arteries or with occlusion of fewer than four vessels since these techniques are commonly used in stroke models. Studies that did not use selective Src kinase inhibitors or studies in which the intervention did not directly result in Src kinase modulation were not eligible for inclusion.

### Risk of bias assessment

Assessment of risk for bias was based on the Systematic Review Center for Laboratory Animal Experimentation (SYRCLE) Risk of Bias (RoB) tool (Hooijmans et al., 2014), which was derived by the Cochrane Risk of Bias tool. SYRCLE RoB tool consists of nine questions adjusted for the specific characteristics of bias contributing to the results of interventional preclinical studies. Each question was marked as “Yes,” “No,” or “Unclear.”

### Data extraction

From each study, the following data were extracted: authors' names, year of publication, sample size, type of animal model and age, type of Src kinase inhibitor and timing of administration, method for HI induction, Src kinase inhibition outcomes, role of Src kinases (protective/damaging on neuron's survival) and whether reperfusion took place.

## Results

### Eligible studies

**Figure 1:**
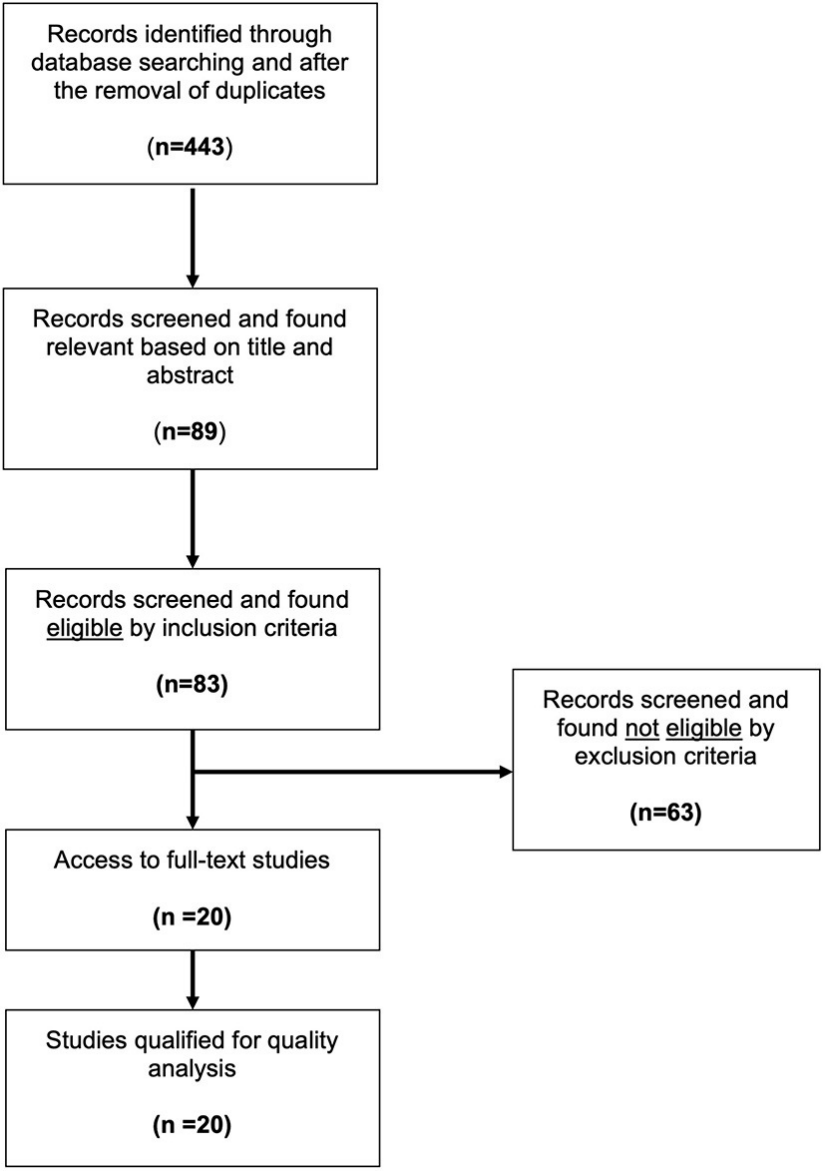
Flow diagram of the literature search. Modified from the preferred reporting items for systematic review and meta-analysis protocols (PRISMA-P) and modified to accurately depict the literature research (Moher et al., 2009).

Results from the Medline database were combined by a citation manager software (Mendeley Desktop, Version 1.19.8), and duplicate entries were removed. Our search yielded 443 studies that were then screened by title and abstract to meet our criteria. Eighty-nine studies were found relevant to this review and required further analysis. Access to full-text articles, despite repeated efforts, was not possible for six studies, thus these were excluded. The remaining eighty-three studies were screened using our pre-defined inclusion and exclusion criteria. Sixty-three studies did not meet our inclusion criteria. Most studies (twenty-one) were excluded due to the use of cell cultures. Other common reasons for exclusion were the lack of an Src kinase inhibitor in the protocol (16 studies) or of a hypoxic event (nine studies). Six studies described unilateral brain hypoxia (mimicking stroke), seven studies were literature reviews and, finally, four articles were published in a non-English language. Table 1 summarizes the reasoning used for exclusion of the sixty-three studies. Ultimately, twenty studies were considered eligible for this analysis. Due to the variability in experimental methods and outcomes, it was impossible to perform a meta-analysis on the effect of Src kinase inhibition on the neonatal brain after hypoxia, so a qualitative approach was taken instead. An illustration of our methodology and approach is demonstrated in Figure 1.

**Table 1:** Studies not eligible based upon the exclusion criteria.

**Exclusion criteria**	**No of studies**
No hypoxic-ischemic brain injury	9
No global (unilateral) ischemia	6
No Src kinase inhibitor	16
Other types of cells/cell lines	21
Non-English	4
Review	7
Overall	63

### Risk for bias assessment

As previously described, the evaluation of risk for bias was based on the SYRCLE RoB tool (Hooijmans et al., 2014). The twenty studies that were included in this review were evaluated for selection, detection, performance, and attrition bias. All but six studies (14/20, 70%) were prone to selection bias because the authors did not adequately describe the methodology used for assigning the animals to the experimental groups. In all twenty studies (20/20, 100%), the investigators were not blinded to the intervention (performance bias), the animals were not housed randomly (performance bias), or the animals were not randomized for outcome assessment (attrition bias). Only two studies (2/20, 10%) had two independent assessors review their results (neuropathology scores) and only two studies (2/20, 10%) specifically described the allocation concealment process. All the studies had animals that were comparable at baseline prior to group assignment, did not selectively present their outcomes, and appropriately addressed incomplete data. The results from the risk for bias analysis are shown in Table 2.

**Table 2:** Assessment of risk of bias based upon SYRCLE RoB tool.

	**Selected Studies**																				
**Risk of bias**	**Type of bias**	**Wang et al. (2004)**	**Guo et al. (2006)**	**Zhang et al. (2007)**	**Wu et al. (2008)**	**Xu et al. (2008)**	**Jiang et al. (2008)**	**Mishra et al. (2009)**	**Hu et al. (2009)**	**Tian et al. (2009)**	**Rehni et al. (2011)**	**Delivoria-Papadopoulos et al. (2011) **	**Delivoria-Papadopoulos (2012) **	**Kumar et al. (2014)**	**Angelis et al. (2014)**	**Angelis et al. (2015)**	**Angelis and Delivoria-Papadopoulos (2017a,b) **	**Angelis and Delivoria-Papadopoulos (2017a,b) **	**Kratimenos et al. (2017) **	**Kratimenos et al. (2018) **	**Kratimenos et al. (2022) **
1 Was the allocation sequence adequately generated and applied?	Selection bias	1	1	1	1	1	1	2	1	1	1	2	1	2	1	1	1	1	2	2	2
2 Were the groups similar at baseline or were they adjusted for confounders in the analysis?	Selection bias	2	2	2	2	2	2	2	2	2	2	2	2	2	2	2	2	2	2	2	2
3 Was the allocation adequately concealed?	Selection bias	1	1	1	1	1	2	1	1	1	1	1	1	1	1	1	1	1	1	1	2
4 Were the animals randomly housed during the experiment?	Performance bias	1	1	1	1	1	1	1	1	1	1	1	1	1	1	1	1	1	1	1	1
5 Were the caregivers and /or investigators blinded from knowledge which intervention each animal received during the experiment?	Performance bias	1	1	1	1	1	1	1	1	1	1	1	1	1	1	1	1	1	1	1	1
6 Were animals selected at random for outcome assessment?	Detection bias	0	0	0	0	0	0	0	0	0	0	0	0	0	0	0	0	0	0	0	0
7 Was the outcome assessor blinded?	Detection bias	1	1	1	1	1	1	1	1	1	1	1	1	2	1	1	1	1	1	2	1
8 Were incomplete outcome data adequately addressed?	Attrition bias	2	2	2	2	2	2	2	2	2	2	2	2	2	2	2	2	2	2	2	2
9 Are reports of the study free of selective outcome reporting?	Reporting bias	2	2	2	2	2	2	2	2	2	2	2	2	2	2	2	2	2	2	2	2
Overall assessment		**IR**	**IR**	**IR**	**IR**	**IR**	**IR**	**IR**	**IR**	**IR**	**IR**	**IR**	**IR**	**LR**	**IR**	**IR**	**IR**	**IR**	**IR**	**IR**	**IR**
		**11**	**11**	**11**	**11**	**11**	**12**	**12**	**11**	**11**	**11**	**12**	**11**	**13**	**11**	**11**	**11**	**11**	**12**	**13**	**13**
**YES (2)**																					
UNCLEAR/NOT MENTIONED (1)																					
NO (0)																					
Low Risk (13-18)	LR																				
Intermediate Risk (7-12)	IR																				
High Risk (1-6)	HR																				

### Study characteristics

Four different species of animals were used in the included studies: Sprangue-Dawley rats (n_studies_ = 7, 35%), Yorkshire newborn piglets (n_studies_ = 10, 50%), Swiss albino mice (n_studies_ = 2, 10%), and mice CD1 strain (n_studies_ = 1, 5%). Twelve studies (n_studies_ = 12, 60%) specified the total number of animals used; the remaining studies (n_studies_ = 8, 40%) did not document the number of animals and the size of the assigned groups.

In all studies, a single dose of Src inhibitor was administered. In eighteen studies (n_studies_ = 18, 90%) the investigators administered a selective Src kinase inhibitor (PP1, PP2, PP3, or SU6656). The remaining two studies (n_studies_ = 2, 10%) included two different non-selective inhibitors of Src kinase, the neuronal nitric oxide synthase inhibitor (nNOSi) and (*RS*)-2-amino-3-(3-hydroxy-5-*tert*-butylisoxazol-4-yl) propanoic acid (ATPA), which also modulated the activity of the Src kinase after the hypoxic insult. In addition, one study (n_studies_ = , 5%) combined therapeutic hypothermia with Src-i. The Src-i and their characteristics are presented in Supplementary Table 1.

Different models of HI were used in the included studies, primarily depending on the animal species involved. Eleven studies (n_studies_ = 11, 55%) used FiO2 titration to establish transient cerebral hypoxia; most of these studies (10/11, 91%) utilized piglets. In two studies (n_studies_ = 2, 10%), investigators performed bilateral carotid occlusion (2VO) in Swiss albino mice and in seven of them (n_studies_ = 7, 35%) they used the method of 4VO in Sprangue-Dawley rats. According to the data that were extracted, reperfusion ensued after HI in every study. Analyzed studies, the species and number of animals, method of HI and type of Src-i used are summarized in Table 3.

**Table 3:** The included studies and their descriptive statistics.

**No**	**Year**	**Authors**	**Type of animals**	**No of animals**	**Interventions**	**Method of HI**
1	2004	Wang et al., 2004	Adult Sprangue-Dawley rats	-	10 μl PP2 (icv)	4VO
2	2006	Guo et al., 2006	Adult Sprangue-Dawley rats	-	5 μg/μl PP2 (icv), 5 μg/μl PP3 (icv), 5 μg/μl locostatin (icv)	4VO
3	2007	Zhang et al., 2007	Adult Sprangue-Dawley rats	-	1 mg/kg muscimol (ip), 20 mg/kg baclofen (ip), 15 μg PP2 (ip), 15 μg PP3 (ip), 3 mg/kg MK-801 (ip)	4VO
4	2008	Wu et al., 2008	Adult Sprangue-Dawley rats	-	25 μg PP2 (icv)	4VO
5	2008	Xu et al., 2008	Adult Sprangue-Dawley rats	-	2 nmol ATPA in 5 μl of 0.9% NaCl (icv)	4VO
6	2008	Jiang et al., 2008	Newborn mice CD1 strain	-	1 μg/mg PP2 (ip), 1 μg/mg PP3 (ip)	Titration of FiO2
7	2009	Mishra et al., 2009	Newborn Yorkshire piglets	5	0.4 mg/Kg nNOSi (iv)	Titration of FiO2
8	2009	Hu et al., 2009	Adult sprangue-Dawley rats	-	5 μM SU-6656 (icv)	4VO
9	2009	Tian et al., 2009	Adult sprangue-Dawley rats	-	100 pmol/animal SU-6656 (icv),	4VO
10	2011	Rehni et al., 2011	Adult swiss albino mice	28	0.1 mg/kg and 0.2 mg/kg PP1 (ip), 2 mg/kg and 4 mg/kg SU-6656 (ip)	2VO
11	2011	Delivoria-Papadopoulos et al., 2011	Newborn Yorkshire piglets	5	1 mg/kg PP2 (iv)	Titration of FiO2
12	2012	Delivoria-Papadopoulos, 2012	Newborn Yorkshire piglets	5	0.4 mg/kg PP2 (iv)	Titration of FiO2
13	2014	Kumar et al., 2014	Adult swiss albino mice	48	0.1 mg/kg and 0.2 mg/kg PP1 (ip), 2 mg/kg and 4 mg/kg SU-6656 (ip)	2VO
14	2014	Angelis et al., 2014	Newborn Yorkshire piglets	5	1 mg/kg PP2 (iv)	Titration of FiO2
15	2015	Angelis et al., 2015	Newborn Yorkshire piglets	4	1 mg/kg PP2 (iv)	Titration of FiO2
16	2017	Angelis and Delivoria-Papadopoulos, 2017a	Newborn Yorkshire piglets	5	1 mg/kg PP2 (iv)	Titration of FiO2
17	2017	Angelis and Delivoria-Papadopoulos, 2017b	Newborn Yorkshire piglets	5	1 mg/kg PP2 (iv)	Titration of FiO2
18	2017	Kratimenos et al., 2017	Newborn Yorkshire piglets	5	1 mg/kg PP2 (iv)	Titration of FiO2
19	2018	Kratimenos et al., 2018	Newborn Yorkshire piglets	5	PP2 and hypothermia	Titration of FiO2
20	2022	Kratimenos et al., 2022	Newborn Yorkshire piglets	5	1 mg/kg PP2 (iv)	Titration of FiO2

iv, intravenous; icv, into the cerebral ventricle; ip, intra-peritoneal; mg, milligram; kg, kilogram.

Concerning the timing of intervention, the experimental compound was administered after the onset of the HI event in only three of the twenty studies (n_studies_ = 3, 15%), mimicking the actual sequence of events in clinical practice. In the remaining seventeen studies (n_studies_ = 17, 85%), the compound was given prior to the induction of the hypoxic insult. Two studies (n_studies_ = 2, 10%) described conditioning which entails several brief repetitive cycles of ischemia with intermittent reperfusion prior to or subsequently to prolonged ischemia (Rehni et al., 2011; Kumar et al., 2014). Both studies demonstrated the neuroprotective properties of the Src kinase under HI conditions. It is worth mentioning that studies (n_studies_ = 12, 60%) that focused exclusively on biochemical analyses did not include a sham or control group for direct comparisons (treated vs. non-treated) of the effectiveness of the therapy with Src-i.

The inclusion of eligible studies led to the assessment of histologic, biochemical, and neurobehavioral outcomes. Histologic analyses included cortical and striatal lesions. Moreover, biochemical parameters, such as the enzymatic expression or activity related to neurological damage, and the cerebral energy status as quantified by ATP and phosphocreatine (PCr) concentrations, were evaluated. In ten out of twenty studies (n_studies_ = 10, 50%) sufficient cerebral hypoxia was induced for both the treatment and control groups as confirmed by energy production levels. Neurobehavioral indicators were examined with neurobehavioral tests designed to measure cognitive function (memory) and motor coordination. Two studies (n_studies_ = 2, 10%) included both histologic and neurobehavioral outcomes, eleven studies (n_studies_ = 11, 55%) only biochemical outcomes and six (n_studies_ = 6, 30%) reported both biochemical and histologic outcomes. It is worth mentioning that one study (n_studies_ = 1, 5%) utilized experimental data to create and validate a computational model of the critical intracellular signaling components of HI in neonatal brain (Kratimenos et al., 2022).

Of the studies included in our analysis, six (n_studies_ = 6, 30%) demonstrated that Src kinase phosphorylation was neuroprotective, whereas 14 studies (n_studies_ = 14, 70%) provided evidence that the effects of Src kinase can be deleterious while its inhibition provides neuroprotection. Six out of twenty studies (n_studies_ = 6, 30%), where Src kinase activity had a beneficial impact, were conducted on adult animal models. The studies in which Src kinase activity led to worse outcomes were conducted on either newborn animals (n_studies_ = 9, 45%) or adult animals (n_studies_ = 5, 25%). The time of intervention, type of outcome measures, and the role of Src kinase in selected studies are presented in Table 4.

**Table 4:** The included studies and their descriptive statistics.

**No**	**Year**	**Authors**	**Time of intervention**	**Type of outcomes**	**Outcomes with Src kinase inhibition**	**Role of Src**
1	2004	Wang et al., 2004	Pre HI	Biochemical	Reduced ERK5	Protective
2	2006	Guo et al., 2006	Pre HI	Biochemical	Reduced Ras/Raf-1/ERK	Protective
3	2007	Zhang et al., 2007	Pre HI	Biochemical	Reduced NR2A, PSD-95, Src, increased GABA	Deleterious
				Histologic	Reduced neuronal injury	
4	2008	Wu et al., 2008	Pre HI	Biochemical	Reduced Spry2	Deleterious
5	2008	Xu et al., 2008	Pre HI	Biochemical	Reduced NR2A, PSD-95, Src	Deleterious
				Histologic	Reduced neuronal injury	
6	2008	Jiang et al., 2008	Post HI	Biochemical	Reduced Src, NR2A, NR2B, unchanged PDS95	Deleterious
				Histologic	Reduced neuronal injury	
7	2009	Mishra et al., 2009	Pre HI	Biochemical	Increased ATP, PCr	deleterious
					Reduced Src kinase	
8	2009	Hu et al., 2009	Pre HI	Biochemical	Reduced ERK, Era, CREB	Protective
					Increased PP2A	
9	2009	Tian et al., 2009	Pre HI	Biochemical	Reduced ERK	Protective
				Histologic	Reduced neuronal injury	
10	2011	Rehni et al., 2011	Pre HI	Histologic	Increased infarct size	Protective
				Neurobehavioral	Impaired memory (elevated plus maze test)	
					Motor incoordination (rota-rod test)	
11	2011	Delivoria-Papadopoulos et al., 2011	Pre HI	Biochemical	Increased ATP, PCr	Deleterious
					Reduced CaM, CaM kinase IV, CREB	
12	2012	Delivoria-Papadopoulos, 2012	Pre HI	Biochemical	Increased ATP, PCr	Deleterious
					Reduced caspase-3/-9	
13	2014	Kumar et al., 2014	Pre HI	Histologic	Increased infarct size	Protective
				Neurobehavioral	Impaired memory (Morris-water-maze test)	
					Motor incoordination (rota-rod test)	
					Motor incoordination (inclined beam walking)	
					Motor incoordination (lateral push test)	
14	2014	Angelis et al., 2014	Pre HI	Biochemical	Increased ATP, PCr	Deleterious
					Reduced both caspase-1, IL-1β	
15	2015	Angelis et al., 2015	Pre HI	Biochemical	Increased ATP, PCr	Deleterious
					Reduced caspase-1/-8	
16	2017	Angelis and Delivoria-Papadopoulos, 2017a	Pre HI	Biochemical	Increased ATP, PCr	Deleterious
					Reduced PTP-1B	
17	2017	Angelis and Delivoria-Papadopoulos, 2017b	Pre HI	Biochemical	Increased ATP, PCr	Deleterious
					Reduced caspase-2	
18	2017	Kratimenos et al., 2018	Post HI	Biochemical	Increased ATP, PCr	Deleterious
					CaM kinase IV, additive effect of hypothermia	
				Histologic	Reduced neuronal injury	
19	2018	Kratimenos et al., 2017	Pre HI	Biochemical	Increased ATP, PCr	Deleterious
					Reduced caspase-3, cytochrome c, smac/diablo, AIF	
				Histologic	Reduced neuronal injury	
20	2022	Kratimenos et al., 2022	Post-HI	Biochemical	Increased ATP, PCr	Deleterious
					Increased Ca^2+^ influx, CaMKK2	
				Computational model	Ca^2+^ influx and Bax expression are dissociable	
					Reduced Bax expression by altering NMDAR – Src kinase interaction	

## Discussion

This paper highlights the current evidence on neuroprotective effects of Src kinase modulation as demonstrated by histologic, biochemical, and neurobehavioral outcomes in twenty eligible studies. Notably, all the included studies were based on a single dose regimen, but only 15% of the studies administered Src-i post-HI. This treatment timing is a key consideration, as in actual clinical practice, treatment with Src-i would likewise occur post-HI.

Although various animal models were utilized in the studies included, all inhibitors used were selective for Src kinase. Each preclinical study that examined models of the neonatal age group reported neuroprotective effects of treatment with Src-i, whereas studies in adult rats showed the opposite effect. This discrepancy may be attributed to the pathophysiologic differences between neonatal and adult brains as it pertains to susceptibility to injury, plasticity and cell death pathway activation (Sands et al., 1979; Clancy et al., 2007; Pressler and Auvin, 2013).

Methodological quality assessment using the SYRCLE's RoB tool yielded an intermediate risk for bias scores for the evaluated studies in this review. Due to the variability of experimental methods and outcomes used, it was not possible to perform a meta-analysis on the effect of Src kinase inhibition on the neonatal brain after hypoxia.

### Pathophysiology of HI in neonatal brain

**Figure 2:**
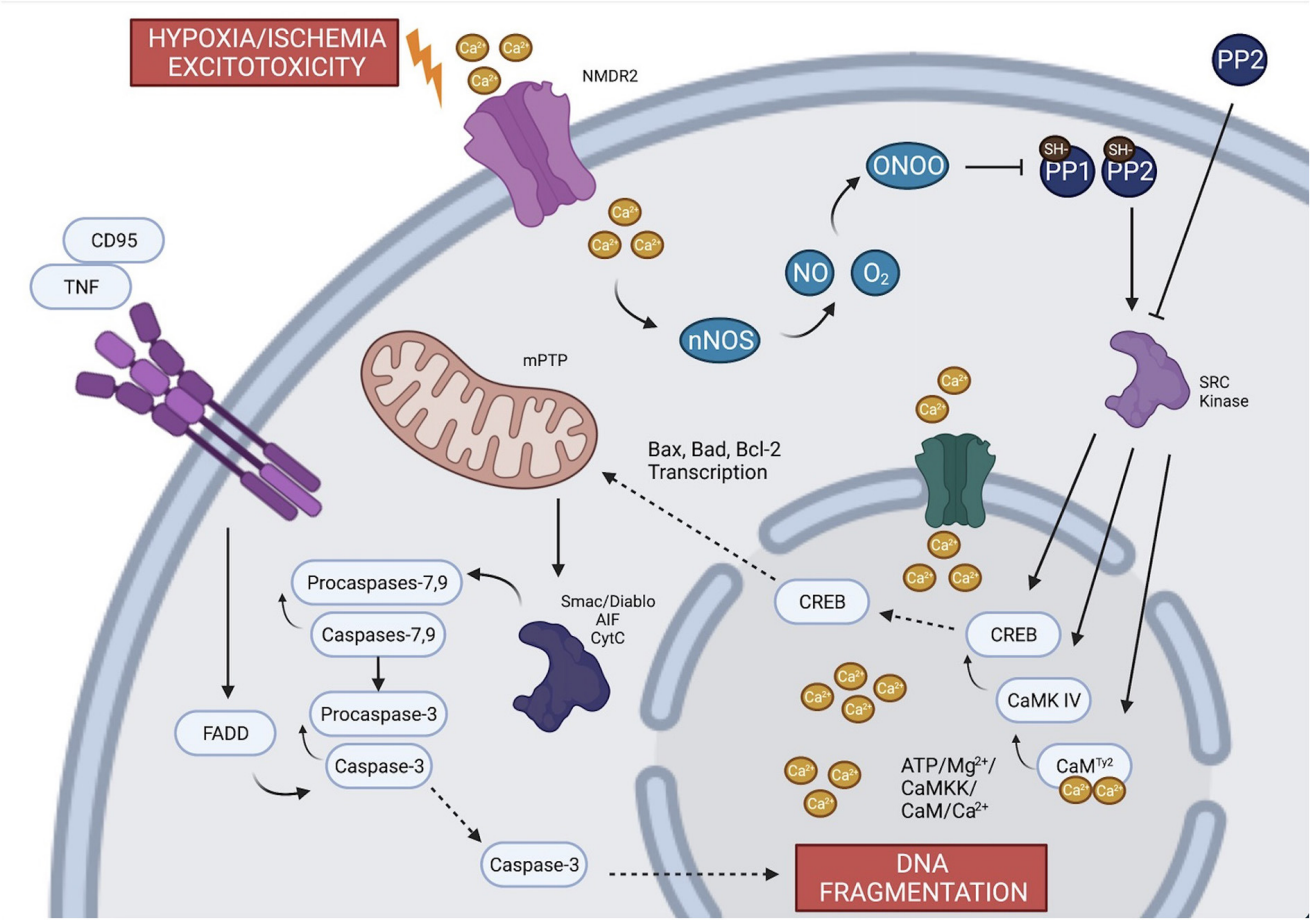
The proposed mechanism of the apoptotic pathway in a developing neuron. During global ischemia, evidence suggests that the three major mechanisms of neuronal cell death (excitoxicity, generation of free radicals and inflammation) are mediated through the phosphorylation of regulatory sites by Src kinases. Failure of the oxidative phosphorylation gives rise to intracellular Ca^2+^ which triggers ecotoxicity and potentiates calcium-dependent apoptotic pathways. Calcium contributes to the generation of free radicals through NO and nNOS and leads to transcription of factors that induces leakage of apoptotic proteins from mitochondria. The inflammation and the extrinsic apoptotic pathway are mediated through TNF and different types of caspases. Created with BioRender.com.

There are three major mechanisms of neuronal cell death during global ischemia: generation of free radicals, excitotoxicity, and inflammation. Each mechanism is mediated through inflammatory cascades that require phosphorylation of enzymatic regulatory sites by Src kinases (Mishra et al., 2009; Delivoria-Papadopoulos et al., 2011; Delivoria-Papadopoulos, 2012; Angelis et al., 2014, 2015; Angelis and Delivoria-Papadopoulos, 2017a,b; Kratimenos et al., 2017, 2018). Aligned with these findings, experiments on newborn piglets demonstrated that inhibition of Src kinase phosphorylation after HI by a selective antagonist (Src-i) is a novel mechanism of neuroprotection (Mishra et al., 2009; Delivoria-Papadopoulos et al., 2011; Delivoria-Papadopoulos, 2012; Angelis et al., 2014, 2015; Angelis and Delivoria-Papadopoulos, 2017a; Kratimenos et al., 2017, 2018). The proposed mechanism of the apoptosis-induced cell death in a developing neuron is illustrated in Figure 2.

The activation of Src kinases in HI is dependent, in part, upon formation of free radicals, which are known to develop during HI in the setting of decreased oxidative phosphorylation given decreased O2. Nitric oxide (NO) free radicals, which are produced by neuronal nitric oxide synthase (nNOS), react with superoxide to form peroxynitrate. Peroxynitrate then inactivates protein tyrosine phosphatases, SH-PTP-1 and SH-PTP-2 *via* reduction mechanisms on cysteine residues (Lee et al., 1998; Barrett et al., 1999; Takakura et al., 1999), facilitating the activation of Src kinases (Mishra et al., 2009).

In addition to the generation of free radicals as oxidative phosphorylation fails, this metabolic alteration also induces excitotoxicity through the depolarization and activation of voltage-gated Ca^2+^-channels with a subsequent rise in intracellular Ca^2+^ (White et al., 2000). Depolarization of those channels results in the release of excitatory neurotransmitters such as glutamate into the synaptic cleft (Fujimoto et al., 2004) and inability of the glutamate reuptake mechanisms to clear glutamate from the cleft. This cascade of events leads to the upregulation of gated NMDARs, further increasing intracellular Ca^2+^ and activating calcium-dependent apoptotic pathways (excitotoxicity) (Arundine and Tymianski, 2004; Delivoria-Papadopoulos et al., 2011). Additionally, excitotoxicity is augmented by the concurrent phosphorylation of certain subunits of NMDAR by Src kinase (Chen et al., 2003; Salter and Kalia, 2004).

Calcium itself plays a critical role in HI-mediated neuronal cell injury (Delivoria-Papadopoulos et al., 2007). During hypoxia, nuclear Ca^2+^ forms a complex with calmodulin (CaM), which activates an apoptotic cascade (Delivoria-Papadopoulos et al., 2011). This cascade involves Ca^2+^-dependent kinases such as the CaM kinase-dependent kinase (CaMKK) and CaM kinase IV (CaMKIV), primarily located in the neuronal cell nucleus. CaMKK directly activates CaMKIV *via* phosphorylation of threonine 200 (Thr200) or threonine 196 (Thr196) in a process that requires Ca^2+^/CaM and ATP/Mg^2+^. Activated CaMKIV mediates DNA transcription by phosphorylating cyclic AMP response binding protein (CREB) (Mishra et al., 2006; Delivoria-Papadopoulos et al., 2007, 2011; Hornick et al., 2007). During HI, Src kinase phosphorylates CaM at tyrosine 99 (Tyr99), CaMKIV at Thr200 or Thr196 and CREB protein at Ser133, promoting the expression of pro-apoptotic proteins (Delivoria-Papadopoulos et al., 2011). As discussed, inhibition of Src kinase by a potent selective inhibitor has been shown to ameliorate the impact of HI in the cerebral cortex of newborn piglets (Delivoria-Papadopoulos et al., 2011).

Programmed cell death occurs through two pathways: intrinsic and extrinsic. The intrinsic pathway is mediated by the generation of free radicals (oxidative stress) and caspases, whereas the extrinsic pathway is mediated by inflammation and tumor necrosis factor (TNF) (Bleicken et al., 2013; Lukyanova and Kirova, 2015). The Bcl-2 family protein includes several anti-apoptotic (Bcl-2, Bcl-xL, and Bcl-w) and pro-apoptotic (Bax, BAD, Bak, or Bok) proteins, whose transcription is induced by HI (Bleicken et al., 2013; Lukyanova and Kirova, 2015). Activation of the CREB protein leads to apoptosis by the transcription of Bax and suppression of Bcl-2 (Kratimenos et al., 2017). Porcine animal models have shown that activated Bax forms pores in the mitochondrial membrane, allowing the leakage of apoptosis-inducing factor (AIF), Smac/Diablo and cytochrome c in the cytosol (Kratimenos et al., 2017). The release of these molecules is further facilitated by the action of Src kinases that expand the opening of the mitochondrial permeability transition pore (mPTP) (Kratimenos et al., 2017). The translocation of apoptotic factors activates caspases −3, −7, and −9, which results in DNA fragmentation and neuronal cell death (Kratimenos et al., 2017). These caspases mediate the activation of the intrinsic apoptotic pathway, whereas the extrinsic pathway is activated by TNF through the binding of Fas ligand (CD95L) to the CD95 receptor. This process results in the formation of the complex FAS-associated death domain (FADD) which then activates caspase-8 (Boatright et al., 2003). Caspase-8 induces apoptosis *via* caspase-3 activity. Additionally, *in vivo* studies have demonstrated the inhibitory effect of Protein Phosphatase 2 (PP2) on caspase-8, which would in this case ameliorate the observed apoptosis (Angelis et al., 2015).

Research indicates that activity of both caspase-8 and caspase-1 acutely increases in the newborn piglet brain following hypoxia (Angelis et al., 2015). Capsase-1 activation is directly related to neuro-inflammation and contributes to the production of IL-1β through the formation of inflammasomes (Angelis et al., 2015). Furthermore, Src kinase inhibition protects cortical neurons from the deleterious sequelae of inflammation caused by HI (Angelis et al., 2015). Additionally, there is evidence that Src kinase is involved in apoptotic cascade activation and pro-neuroinflammatory pathways, ultimately leading to neuronal cell death (Angelis et al., 2015).

### N-methyl-D-aspartate receptors (NMDARs) in HI

The balance between neuronal inhibition and excitation is an essential homeostatic mechanism for typical brain function and development. HI disrupts this homeostasis by upregulating excitation, resulting in apoptosis and neuronal cell death (Seeburg, 1993; Hollmann and Heinemann, 1994). The excitatory component of this mechanism is mediated by glutamate and the NMDARs (Kumari and Ticku, 2000). NMDARs and their associated signaling pathways are located at the electron-dense matrix beneath the postsynaptic membrane of excitatory synapses, called postsynaptic density (PSD) (Kennedy, 1997; Martone et al., 1999). HI-induced activation of Src kinases upregulates NMDARs, increasing excitation by phosphorylating tyrosine residues on the NR2A and NR2B subunits. In addition, transient ischemia causes changes in the structure and protein composition of the PSD, enhancing their association with certain proteins (Kennedy, 1997; Martone et al., 1999).

The interaction between NR2A, Src kinase and post-synaptic density protein 95 (PSD-95) has been implicated in HI-induced neuronal injury. Liu et al. suggest that this mechanism also includes the activation proline-rich kinase 2 (Pyk2). After ischemia-reperfusion (I/R), activated Pyk2 binds to Src kinase, promoting phosphorylation of NR2A and calcium overload via PSD-95 (Liu et al., 2005). PP2 can block the NR2A-PSD95-Src signaling pathway and alleviate neuronal cell injury (Zhang et al., 2007; Jiang et al., 2008). Other than PP2, MK-801, a selective antagonist of NMDARs, can reverse Src kinase's activation and its effect on NR2A during HI. A novel approach using a computational model also predicted that Src-i can modulate the interaction between the NMDARs and Src and can significantly reduce Bax expression (39).

In the setting of HI-induced excitotoxicity, targeting gamma-aminobutyric acid (GABA) signaling is a potentially effective therapeutic strategy. GABA is the primary inhibitory neurotransmitter in the CNS that balances the excitatory effects of glutamate (Oja et al., 1990; Rosenbaum et al., 1990; Johansen and Diemer, 1991; Sivilotti and Nistri, 1991). Several researchers have proposed that enhancing GABAergic activity could potentially alleviate the excitotoxic effects of ischemic brain injury (Oja et al., 1990; Rosenbaum et al., 1990; Johansen and Diemer, 1991; Sivilotti and Nistri, 1991). The effects of GABA on ischemia are mediated by the activation of GABA_A_, which increases Cl^−^ permeability and hyperpolarizes cells. Hyperpolarized cells demonstrate reduced excitability due to decreased glutamate concentrations and calcium influx (Oja et al., 1990; Rosenbaum et al., 1990; Johansen and Diemer, 1991; Sivilotti and Nistri, 1991). Furthermore, Zhang et al. demonstrated that muscimol and baclofen, both GABA receptor agonists, prevent hippocampal CA1 neurons' death during cerebral I/R via suppression of the phosphorylation of excitatory NMDA receptor subunit NR2A (Zhang et al., 2007). Interestingly, muscimol's and baclofen's neuroprotective properties are linked to the downregulation of the phosphorylation of Src kinase and NMDARs. In addition, administration of (RS)-alpha-amino-3-hydroxy-5-tert-butyl-4-isoxazolepropionic acid (ATPA), an agonist of GluR5 (glutamate receptor 5)-containing kainate receptor, also demonstrated neuroprotective effects (Zhang et al., 2007). Xu et al. hypothesized that this was secondary to increased GABA release and inhibition of the NR2A-PSD95-Src signaling pathway (Xu et al., 2008). However, some researchers have also reported conflicting results, indicating that increased GABA signaling after HI may accelerate neuronal cell loss (Rosenbaum et al., 1990; Stokes et al., 2001).

### The dual role of Src kinase following ischemia-reperfusion (I/R)

Several studies have highlighted Src kinase's role in neuronal survival after I/R through interactions with the extracellular signal-regulated kinase (ERK) (Wang et al., 2004; Guo et al., 2006; Wen et al., 2008; Wu et al., 2008; Hu et al., 2009; Tian et al., 2009). Following I/R, Src kinase and NMDARs upregulate ERK, increasing neuronal survival (Wang et al., 2004; Guo et al., 2006). HI-induced activation of Src kinase leads to the phosphorylation of Raf at the Tyr340/341 position. Raf-1, an upstream molecule of the ERK pathway, subsequently induces the phosphorylation of estrogen receptor a (ERa) and CREB at Ser133 position, promoting neuronal cell survival (Wang et al., 2004; Guo et al., 2006; Wu et al., 2008; Hu et al., 2009). Despite some reports of Src kinase-related increases in neuronal survival, several studies showed that Src kinase inhibition by PP2A was neuroprotective following I/R. Wu et al. demonstrated that Src kinase's induction of neuronal apoptosis following I/R, is mediated by the phosphorylation of Spry2, a down-regulator of Raf/ERK pathway (Wu et al., 2008). Administration of PP2 or SU6656 shows an attenuation of Src kinase's negative effect on cellular death in rat hippocampi (Wang et al., 2004; Guo et al., 2006; Hu et al., 2009; Tian et al., 2009). The results of the aforementioned studies cannot be translated to clinical neonatology practice because of the use of adult mice and their differences in pathophysiology, mainly on the mechanism of injury and recovery when compared to neonatal mice (Sands et al., 1979; Clancy et al., 2007; Pressler and Auvin, 2013). Moreover, intact Src kinase is correlated with improved neuronal survival, whereas in conditions of excitotoxicity, calpain cleavage of Src kinase generates a neurotoxic truncated Src fragment (Hossain et al., 2015).

### Pre-/post-conditioning

As mentioned previously, Src kinase plays a pivotal role in neuronal health among multiple disease models, including conditioning. Conditioning involves the intermittent reperfusion that precedes or follows prolonged ischemia. These are termed ischemic preconditioning (IPrCo) and ischemic post-conditioning (IPoCo), respectively (Kumar et al., 2014). Studies have shown that both IPrCo and IPoCo prevent cerebral infarct formation by ischemia-reperfusion and prevent neurobehavioral impairment in Swiss albino mice (Rehni et al., 2008a, 2011; Kumar et al., 2014). IPrCo's neuroprotective effect on the brain is likely attributable to amelioration of the ischemia and reperfusion sequela through the activation of the Akt/p38-mitogen/ERK pathway (Bochelen et al., 1999; Rehni et al., 2008b, 2009; Kumar et al., 2014). However, Rehni et al. demonstrated that the effect of IPrCo is exerted through phosphorylation of Src kinase, even though the exact activation transduction pathway is not yet well understood (Rehni et al., 2008a, 2011). Neuronal cell injury was also ameliorated by IPoCo through the activation of Src kinase (Kumar et al., 2014). Although the exact mechanism remains unknown, Kumar et al. suggested that it also involves the activation of Akt/p38-mitogen/ERK (Kumar et al., 2014). In a clinical setting, IPrCo is not feasible due to the inability to predict the onset of ischemia. IPoCo, on the other hand, is clinically relevant.

### Why are findings for Src-i not yet translatable?

Although Src kinase inhibition exhibits a neuroprotective effect on neonatal animal models, this has not yet been confirmed in clinical trials. The variability of experimental results and animal models used has prevented the introduction of Src kinase inhibition as a therapeutic approach in clinical trials. Neurobehavioral experiments could provide additional data to support the use of Src-i, however they require a longer follow-up period with highly trained personnel, as well as validated scoring systems for accuracy and consistency. In the present analysis, only two studies provided a complete set of outcomes that were evaluated with multiple different approaches (Rehni et al., 2011; Kumar et al., 2014). The use of histologic and biochemical markers has been proven to be a cost-effective alternative approach. Further investigations are required to elucidate the precise mechanism by which Src kinase affect cortical neurons. Many Src kinase inhibitors like dasatinib are currently being tested as adjuvant therapies in cancer. Pharmacokinetic data of such inhibitors including PP2, a more selective Src kinase inhibitor used in thirteen out of the 20 included studies, are still lacking. Finally, the potential addition of therapeutic hypothermia to Src-i has not been sufficiently explored.

### Future directions

To date, Src-i have only been examined in clinical trials for cancer and neurodegenerative conditions such as Alzheimer's and Parkinson's disease (ClinicalTrials.gov Identifier: NCT00779389, NCT02167256, and NCT03661125). We anticipate that further research will involve large animals and primates. Large animal studies, although expensive, offer the greatest potential of translation to humans, due to the similarities in brain size, gray/white matter ratio, developmental ages and morphology, as well as the localization of injury after HI (Odden et al., 1989; Thoresen et al., 1996; Haaland et al., 1997; Björkman et al., 2006). Additionally, larger sample sizes can help to decrease bias and improve study validity. Standard reporting methods for preclinical studies focused on Src-i are also necessary to minimize reporting bias. Future work needs to focus on HI pathophysiologic mechanisms and Src-i dosage, timing, route of administration, and potential adverse events. Moreover, as therapeutic hypothermia is considered standard of care for HI in neonates, the additive effects of a combined hypothermia/Src kinase inhibition protocol should be further investigated (Kratimenos et al., 2018). Recently, our team validated a computational model with experimental measurements of critical intracellular signaling components and captured key molecular trends in this pathway (Kratimenos et al., 2022). Our computational model indicated that Src-i disassociates Ca^2+^ influx from Bax expression and modulates the interaction between the NMDAR and Src reducing Bax expression (Kratimenos et al., 2022). This model could provide a translational platform to design and screen drugs in neonatal hypoxic brain (Kratimenos et al., 2022).

### Strengths and limitations of the study

To our knowledge, this is the first attempt to systematically evaluate the current literature on preclinical evidence supporting the use of Src kinase inhibitors in models of HI. Notably, we included ten studies in which large animals, including Yorkshire newborn piglets, were treated with Src-i following HI. Yorkshire newborn piglets' brains share many characteristics with the human brain (Odden et al., 1989; Thoresen et al., 1996; Haaland et al., 1997; Björkman et al., 2006). Human neonates suffer from somatosensory cortical and basal ganglia damage after perinatal asphyxia, which has many similarities to findings in term piglets aged 1–5 days after similar insults (Thoresen et al., 1996).

Although every effort was made for a thorough literature search, it is possible that some relevant studies were missed. It was not feasible to perform a quantitative analysis (meta-analysis) of studies with a focus on Src-i in neonatal HI as there was a significant variability in experimental animals used, sample size and reported outcomes (histologic, biochemical, neurobehavioral). Most of those studies were using inhibitors of the Src kinase to investigate mechanistic questions rather than examining its role as a therapeutic target. The lack of neurobehavioral assessments did not allow for the study of HI-induced visual, motor, and cognitive impairments. Moreover, we are unable to examine the clinical safety of Src-i due to the lack of long term follow-up. Most of the studies were characterized as intermediate when assessed for risk for bias, which can be attributed to the insufficient description of experimental methods, protocols and interventions as evaluated by the SYRCLE's RoB tool. Following the ARRIVE (Animal Research: Reporting of *in vivo* Experiments) guidelines could have improved the reporting of results by minimizing publication bias (Hooijmans et al., 2014). However, these guidelines were not published prior to June 2010, and many studies that were included in our review were conducted before that time. Not all studies explicitly added control groups to compare effectiveness against the treatment group again likely because the studies were not designed to examine therapeutic effects. Despite the moderate quality assigned to the examined studies by the SYRCLE RoB tool, the evidence presented still indicates the potential benefits of Src kinase inhibition in neonates suffering perinatal asphyxia.

## Conclusions

This systematic review demonstrates that inhibition of Src during hypoxia-ischemia results in neuroprotection. However, these protective properties were assessed based on varying animal models, study designs, and intervention characteristics. Further preclinical studies on large animals and specific experimental models are required to examine the pharmacokinetics of Src-i and its exact role in programmed neuronal death. While heterogeneity and risk for bias were limiting factors, the overall results indicate that Src-i neuroprotective properties could be a promising therapeutic strategy to neonates after hypoxic events.

## Data availability statement

The original contributions presented in the study are included in the article/Supplementary material, further inquiries can be directed to the corresponding author/s.

## Author contributions

PK and PC conceptualized the manuscript. PC wrote the manuscript with contribution from IK, AV, JG, and SP. PK, BS, and IK edited the manuscript. All authors have read and approved the final manuscript.

## Funding

This work was funded by K12HD001399 (NIH/NICHD) Child Health Research Career Development Award (CHRCDA) (PI: PK), Children's National Board of Visitors Grant (PI: PK), and K12HD001399-20 (NIH/NICHD) Child Health Research Career Development Award (CHRCDA) (PI: IK).

## Conflict of interest

The authors declare that the research was conducted in the absence of any commercial or financial relationships that could be construed as a potential conflict of interest.

## Publisher's note

All claims expressed in this article are solely those of the authors and do not necessarily represent those of their affiliated organizations, or those of the publisher, the editors and the reviewers. Any product that may be evaluated in this article, or claim that may be made by its manufacturer, is not guaranteed or endorsed by the publisher.
