# Supplementary material for: Neuroprotective effect of Src kinase in hypoxia-ischemia: A systematic review
Source: Front Neurosci. 2022 Nov 24;16:1049655. doi: 10.3389/fnins.2022.1049655 (PMC9730728; doi:10.3389/fnins.2022.1049655)
Supplement: Supplementary file 1 [file Table_1.DOCX]

**Supplementary Table 1.** Src kinase inhibitors of the eligible studies and their characteristics

| Src kinase inhibitor | No. of studies | Mechanism of action | Selectivity for Src kinase | Method of administration | Toxicity / Complications |
| --- | --- | --- | --- | --- | --- |
| ATPA | 1 | GluR5 agonist of NMDARs | non-selective | 1x(icv) | Minimal/no data(Santa Cruz Biotechnology, no date) |
| baclofen | 1 | GABA agonist | non-selective | 1x(ip) | Dizziness, weakness, confusion, headache, nausea, constipation, drowsiness, tiredness(Baclofen: MedlinePlus Drug Information, 2019) |
| locostatin | 1 | Irreversible inhibitor of RKIP | non-selective | 1x(icv) | Minimal/no data(Scientific, no date) |
| MK-801 | 1 | NMDAR antagonist | non-selective | 1x(ip) | Schizophrenia-like symptoms, neurotoxicity(Kovacic and Somanathan, 2010) |
| muscimol | 1 | GABA agonist | non-selective | 1x(ip) | Mydriasis, dryness of mouth, ataxia, confusion, euphoria, dizziness, and tiredness(Puschner, 2018) |
| nNOSi | 1 | Inhibitor of nNOS | non-selective | 1x(iv) | Endothelial dysfunction and inflammatory syndrome in patients with autoimmune disease, leading to an escalation of cardiovascular morbidity and mortality(Wong and Lerner, 2015) |
| PP1 | 2 | Src family kinase inhibitor | selective | 2x(ip) | minimal/no data(Chemical, no date a) |
| PP2 | 14 | Src family kinase inhibitor | selective | 8x(iv), 3x(icv), 2x(ip) | Anemia, cough, CNS depression, drowsiness, headache, heart damage, lassitude (weakness, exhaustion), liver damage, narcosis, reproductive effects, teratogenic effects(Chemical, no date b) |
| PP3 | 3 | Inactive analog of PP2 | - | 2x(ip), 1x(icv) | Minimal/no data. Observations in animals suggest it may be irritant(Chemical, no date c) |
| SU-6656 | 4 | ATP-competitive Src family kinase inhibitor | selective | 2x(ip), 2x(icv) | Minimal/no data. Observations in animals suggest it may be irritant(InSolution^TM^ SU6656 - Calbiochem \| 572636, no date) |

*iv: intravenous; icv: into the cerebral ventricle; ip: intra-peritoneal; RKIP: Raf Kinase Inhibitor Protein; NMDARs: N-methyl-D-aspartate Receptors; GABA: gamma-aminobutyric acid; nNOS: neuronal nitric oxide synthase; nNOSi:neuronal nitric oxide synthase inhibitor; CNS: central nervous system*

*Baclofen: MedlinePlus Drug Information* (2019). Available at: https://medlineplus.gov/druginfo/meds/a614022.html%0Ahttps://medlineplus.gov/druginfo/meds/a682816.html%0Ahttps://medlineplus.gov/druginfo/meds/a601209.html%0Ahttps://medlineplus.gov/druginfo/meds/a682530.html (Accessed: 31 October 2022).

Chemical, C. (no date a) *PP1-Safety Data Sheet*. Available at: https://cdn.caymanchem.com/cdn/msds/14244m.pdf.

Chemical, C. (no date b) *PP2-Safety Data Sheet acc. to OSHA HCS*.

Chemical, C. (no date c) *PP3-Safety Data Sheet*.

*InSolution^TM^ SU6656 - Calbiochem | 572636* (no date). Available at: https://www.merckmillipore.com/INTL/en/product/InSolution-SU6656-Calbiochem,EMD_BIO-572636?ReferrerURL=https%3A%2F%2Fwww.google.com%2F&bd=1#anchor_PDS (Accessed: 31 October 2022).

Kovacic, P. and Somanathan, R. (2010) ‘Clinical physiology and mechanism of dizocilpine (MK-801): Electron transfer, radicals, redox metabolites and bioactivity’, *Oxidative Medicine and Cellular Longevity*. Hindawi Limited, 3(1), pp. 13–22. doi: 10.4161/oxim.3.1.10028.

Puschner, B. (2018) ‘Mushroom Toxins’, in *Veterinary Toxicology: Basic and Clinical Principles: Third Edition*. Elsevier, pp. 955–966. doi: 10.1016/B978-0-12-811410-0.00067-2.

Santa Cruz Biotechnology, I. (no date) ‘ATPA-Safety Data Sheet’.

Scientific, T. (no date) ‘Locostatin-Safety Data Sheet’.

Wong, V. C. and Lerner, E. (2015) ‘Nitric oxide inhibition strategies’, *Future Science OA*. Future Medicine Ltd., 1(1). doi: 10.4155/fso.15.35.
